# A dataset for photovoltaic energy potential of the Yaqui Valley, Sonora, México

**DOI:** 10.1016/j.dib.2023.109983

**Published:** 2023-12-16

**Authors:** José Ruelas, Flavio Muñoz, Juan Palomares, José Castro

**Affiliations:** Tecnológico Nacional de México/ ITS de Cajeme, CP, Sonora 85024, México

**Keywords:** Agrivoltaic technology, Photovoltaic potential, Data analysis

## Abstract

The adequate records of climatological variables, especially temperature and radiation, constitute the basis to be able to determine the photovoltaic and agrivoltaic potential of a region, for this purpose, the data collected must be extremely precise, so it is required that they be focused, current and reliable, to have an adequate estimation. This paper presents the dataset used to estimate the photovoltaic potential of the Yaqui Valley, Sonora, México, for agrivoltaic systems, with the objective of determining the photovoltaic energy generation capacity. Specific records of temperature, radiation and humidity variables obtained from 21 meteorological stations distributed in the Yaqui Valley are used to determine the photovoltaic potential in relation to planting surfaces and commercially available agrivoltaic technologies. To do this, the data was filtered, grouped, and normalized according to the ranges of the variables required for the analysis, this data comprise the last three years.

Specifications Table

**Table:** 

Subject	Data Science, Energy.
Specific subject area	Data Science, Big Data Analytics, Renewable Energy, Sustainability and the Environment.
Data format	CVS (Raw data), figure.
Type of data	Table, maps
Data collection	Data were collected from 21 meteorological stations distributed along the south region of the Sonora state in México (www.drryaqui.org.mx). The data were divided in three principal periods. Initially these include date, hour, temperature, rainfall, relative humidity, barometric pressure, solar radiation, wind speed, wind direction, wind speed (gust), dew point. To determine the photovoltaic potential we only need, date, hour, temperature, solar radiation, and relative humidity, for a lapse of time from 5:00 am to 7:30 pm.
Data source location	The Sonora State South is located between the parallels 27°10´39´´; and 27°50´39´´; north latitude, and the meridians 109°55´39´´; and 110°36’39´´, and was obtained from www.drryaqui.org.mx/drrymet/exportar
Data accessibility	Repository name: Replication data for Photovoltaic energy estimation for the Yaqui Valley, Sonora, México. Data identification number: doi:10.7910/DVN/HQALSA Direct URL to data: Replication data for Photovoltaic energy estimation for the Yaqui Valley, Sonora, México. - Harvard Dataverse

## 1 Value of the Data

• This dataset provides climate records over three years for the region that can be used by other researchers to carry out all kinds of statistical analysis.
• Data corresponding to solar radiation, temperature and humidity is required to be accurate, due to the large size of the facilities and the amount of investment, that can generate high expectations in the return of capital, which could not be achieved if there were made an overestimation of the photovoltaic or agrivoltaic capacity.
• The data and records presented in this study involve, in addition to temperature, humidity and solar radiation, records of wind speed and direction, barometric pressure, dew point and rainfall, which can be used by other researchers to carry out all types of statistical analysis.
• This paper presents data and records that can be compared with values derived from satellite observations to analyze their variability. For example, that presented by the National Renewable Energy Laboratory (NREL).
• This dataset is used to determine the photovoltaic potential of the Yaqui Valley region, with which the analysis of the feasibility of implementing agrivoltaic systems can be carried out, for which an analysis of the variables of temperature, radiation, and relative humidity.
• The data originally collected belongs to the Yaqui River Irrigation District, which has 21 meteorological stations strategically distributed in the region, carrying out permanent monitoring. This dataset is publicly accessible.

## 2 Background

This database was created as part of a study whose purpose is to determine the photovoltaic potential, which in turn will allow estimating the agrivoltaic potential of the region [1], [2], [3]. This analysis was done considering the traditional periods of crops in the region and the photovoltaic potential determined using these data. The results of this analysis will allow decisions to be made about economic and energy use in agricultural regions with similar characteristics [4]. Also giving double use to farmland and taking advantage of the photovoltaic potential of the region [5,6]. Where you can combine the double use of land for cultivation, power generation. In addition to the advantage that areas protected from the sun represent for crops [7,8].

## 3 Data Description

**Fig. 1:**
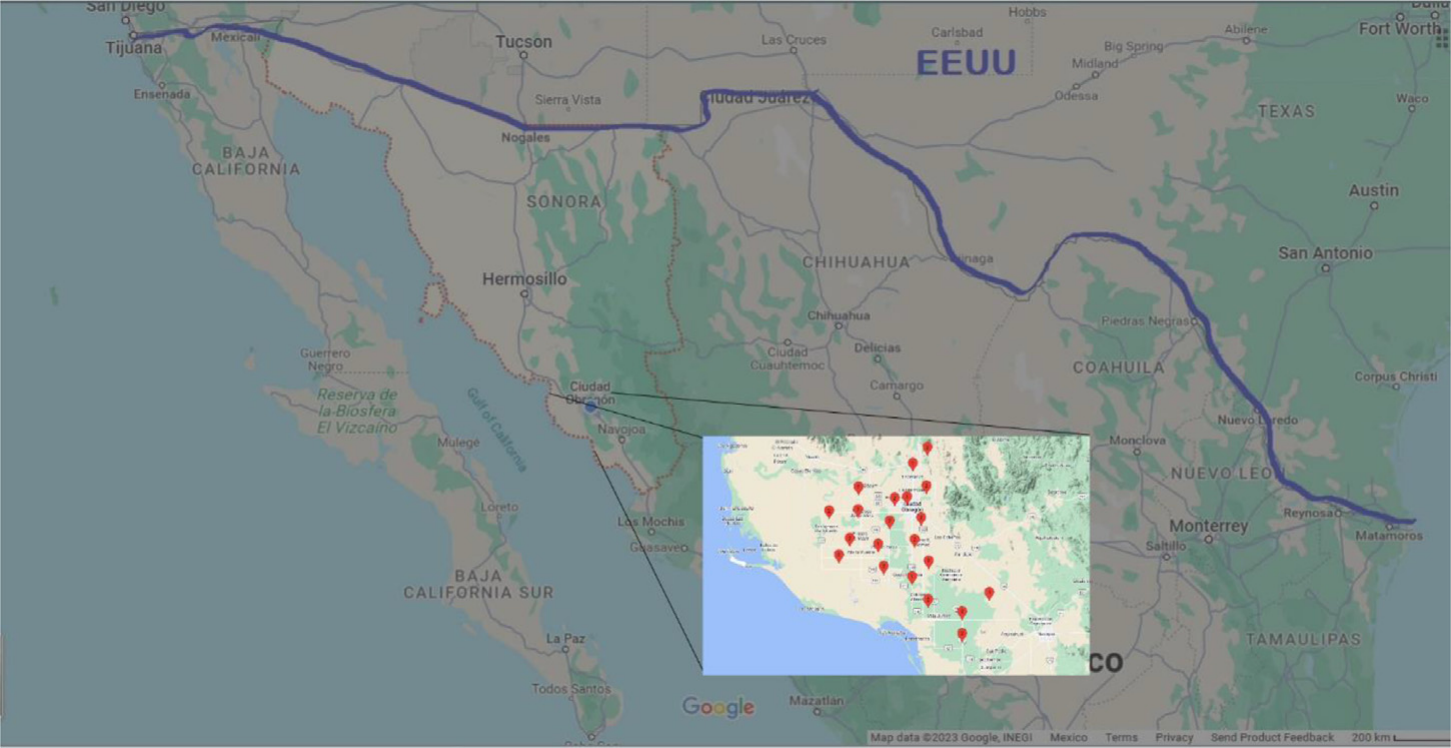
Geographic location of the region (above) and distribution of meteorological stations, taken from google maps - INEGI.

The data was collected from the 21 meteorological stations distributed along the Yaqui Valley Fig. 1, are in turn distributed in 21 compressed files with a .rar extension, within these are the data collected by the weather stations from February 2020 to February 2023, separated into three main periods, from February 22 to June 21, June 22 to October 21 and finally from October 22 to February 21. The data set is divided in these three main periods, according to the traditional planting periods of the Yaqui Valley. This is done for each year included, so in total the .rar file contains 9 folders in csv format, comma separated values, which contain separate information of 11 variables, columns from a to k, indicating date (dd:mm:yy), time (h:m:s), temperature (°C), rain (mm), relative humidity (%), barometric pressure (mmHg), solar radiation (W/m^2^), wind speed (Km/hr), wind direction (^o^N), wind gust (Km/hr), dew point (°C), these Headers are originally displayed in Spanish [9].

## 4 Experimental Design, Materials and Methods

**Fig. 2:**
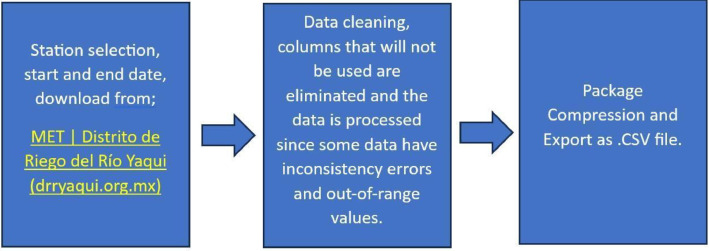
Data preprocessing process.

The database presented in this research was obtained from a public database belonging to the Yaqui River irrigation district (*http://www.dryaqui.org.mx/drrymet/dashboard*), which is continuously monitored through 21 weather stations. The database originally contains 11 variables of which 4 are of interest, date, time, temperature, solar radiation, and relative humidity, so initially the data is filtered, Fig. 2.

**Fig. 3:**
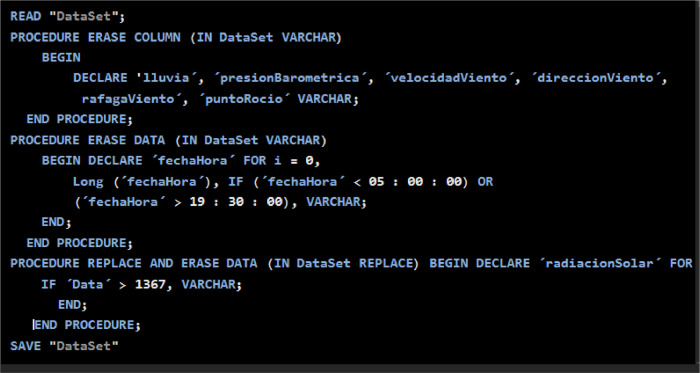
Filtering process pseudocode.

From the original data source, the filtering is carried out in two stages. First, the records are adjusted to a time interval of 5:30 a.m. to 7:30 p.m., because in this range there is significant solar radiation for the analyzed region. Second, filtering is required that data is in the appropriate ranges for the variables that correspond in this case: That is radiation from 0 to 1367 W/m^2^, Fig. 3.

**Fig. 4:**
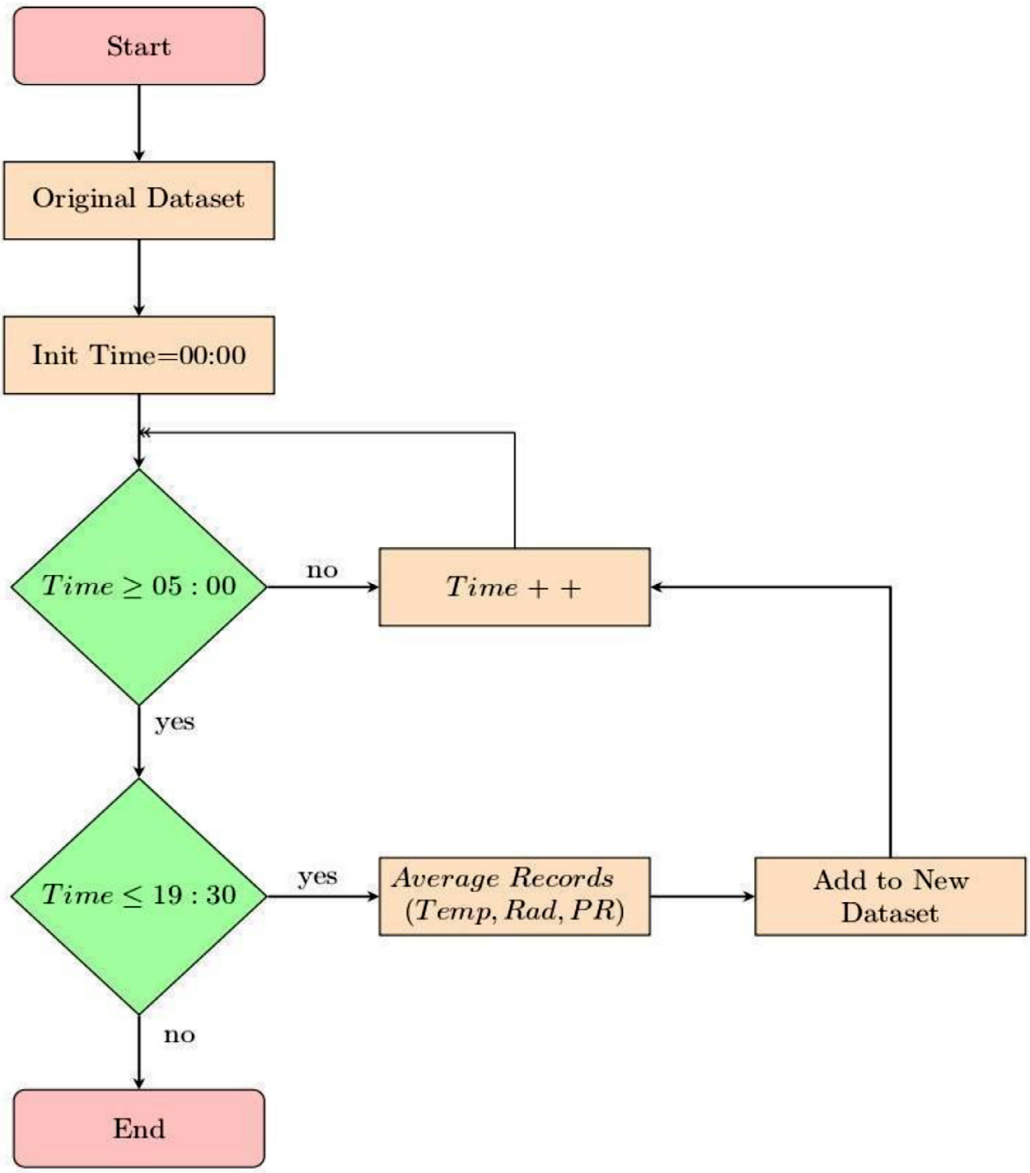
Data pre-processing flow chart.

Once the data has been exported and corrected, a selection is made taking only the hours where there is an acceptable level of solar radiation. Since the measurements of the meteorological stations are not standardized, since they carry out a sample every ten minutes from the start time, these can differ in minutes, so a data selection method was carried out based on the following algorithm, Fig. 4.

**Fig. 5:**
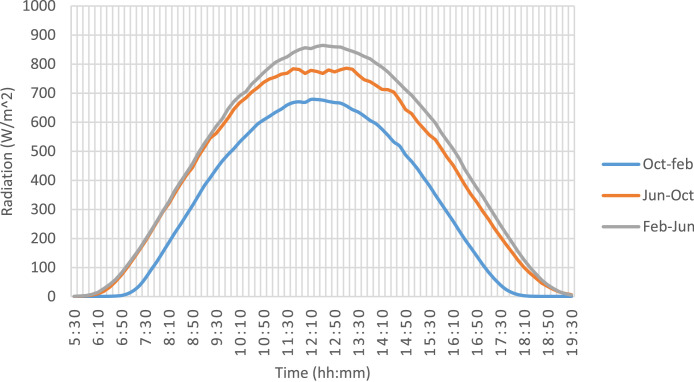
Daily average radiation per period of crops.

For the daily average radiation, Fig. 5. First, it is necessary to establish the average daily radiation available for the region by periods according to the traditional crops of the region where it will be used [9]. The maximum radiation values correspond to the October-February period with values near 900 W/m^2^ and daily radiation values of 5.059 KW/m^2^ (values lower than those reported in the NEREL radiation maps [10], where values are estimated greater than 5.4 KW/m^2^).

**Fig. 6:**
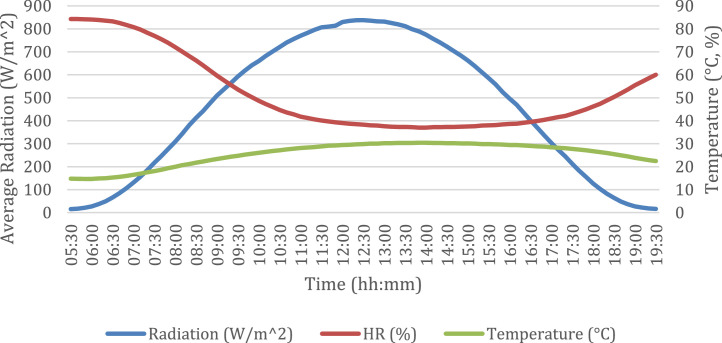
Daily average of solar radiation, relative humidity and temperature.

Finally, with the databases is obtained the daily average of solar radiation, relative humidity and temperature, using for these periods and variants with the main crops of the region for the las 3 years., Fig. 6.

## Limitations

Basically, the limitations consist of the fact that not all the stations worked continuously during the entire period analyzed, some are of more recent installation and others presented some damage on specific days and sometimes anomalous values appear.

## Ethics Statement

The authors declare that we did not conduct human or animal experiments, that we did not gather social media data, and we had (or did not need) permission to use the primary data sources.

## CRediT authorship contribution statement

**José Ruelas:** Conceptualization, Methodology, Validation, Investigation. **Flavio Muñoz:** Conceptualization, Validation, Data curation, Software, Investigation. **Juan Palomares:** Conceptualization, Data curation, Investigation, Validation, Software. **José Castro:** Conceptualization, Data curation, Investigation.

## Data Availability

Replication data for Photovoltaic energy estimation for the Yaqui Valley, Sonora, México (Original data) (Dataverse).
